# Evaluation of the Clinical Outcome and Cost Analysis of Antibiotics in the Treatment of Acute Respiratory Tract Infections in the Emergency Department in Saudi Arabia

**DOI:** 10.3390/antibiotics11111478

**Published:** 2022-10-26

**Authors:** Menyfah Q. Alanazi, Hajar AlQahtani, Thamer A. Almangour, Fadilah Sfouq Aleanizy, Fulwah Yahya Alqahtani

**Affiliations:** 1 Drug Policy & Economic Centre, King Abdulaziz Medical City, Ministry of National Guard-Health Affairs, Riyadh 11426, Saudi Arabia; 2 King Abdullah International Medical Research Centre, Riyadh 11426, Saudi Arabia; 3 King Saud Bin Abdulaziz University for Health Sciences, Riyadh 11426, Saudi Arabia; 4 Department of Pharmaceutical Care, Ministry of National Guard, Health Affairs, Riyadh 11426, Saudi Arabia; 5 Department of Clinical Pharmacy, College of Pharmacy, King Saud University, Riyadh 11451, Saudi Arabia; 6 Department of Pharmaceutics, College of Pharmacy, King Saud University, Riyadh 11495, Saudi Arabia

**Keywords:** respiratory tract infections, antibiotics, appropriate prescribing, cost, emergency department, acute respiratory tract infections

## Abstract

This study aims to assess the prevalence and antibiotic-treatment patterns of respiratory tract infections (RTIs), prevalence and types of antibiotic-prescribing errors, and the cost of inappropriate antibiotic use among emergency department (ED) patients. A cross-sectional study was conducted at the ED in King Abdulaziz Medical City, Riyadh, Saudi Arabia. Patient characteristics (age, sex, weight, allergies, diagnostic tests (CX-Ray), cultures, microorganism types, and prescription characteristics) were studied. During the study, 3185 cases were diagnosed with RTIs: adults (>15 years) 55% and pediatrics (<15 years) 44%. The overall prevalence of RTIs was 21%, differentiated by upper respiratory tract infections (URTI) and lower respiratory tract infections (LRTI) (URTI 13.4%; LRTI 8.4%), of total visits. Three main antibiotics (ATB) categories were prescribed in both age groups: penicillin (pediatrics 43%; adults 26%), cephalosporin (pediatrics 29%; adults 19%), and macrolide (pediatrics 26%; adults 38%). The prevalence of inappropriate ATB prescriptions was 53% (pediatrics 35%; adults 67%). Errors in ATB included selection (3.3%), dosage (22%), frequency (3%), and duration (32%). There is a compelling need to create antimicrobial stewardship (AMS) programs to improve antibiotic use due to the high number of prescriptions in the ED deemed as inappropriate. This will help to prevent unwanted consequences on the patients and the community associated with antibiotic use.

## 1. Introduction

Any infectious disease affecting the upper or lower respiratory tract is referred to as a respiratory tract infection (RTI) [1]. The common cold, laryngitis, pharyngitis/tonsillitis, acute rhinitis, acute rhinosinusitis, and acute otitis media are considered upper respiratory tract infections (URTIs). This includes pediatric, adult, and elderly patients, as well as all subpopulations and both genders [2,3,4]. Frequently, antibiotics are prescribed for RTIs in the emergency department (ED) [5]. Despite RTIs being caused by self-limiting viral illnesses, these infections can cause significant morbidity and, very rarely, mortality. As such, early diagnosis and effective treatment can lower morbidity and substantially overcome mortality.

One of the primary causes of the emergence of antimicrobial resistance (AMR) is excessive and inappropriate use of antibiotics [6,7,8]. Approximately 50% are prescribed for ARTIs against recommended use [2,3,4,7,8]; thus, guidelines have discouraged this practice [2,9]. Inappropriate antibiotic prescriptions lead to an increase in medical care costs [10], encouraging patients to seek further medical attention for the resulting respiratory diseases [11,12]. Patients on antibiotics for a sore throat often visit the hospital again for treatment [12]. This increased use of services creates needless healthcare costs. The cost of antibiotic prescriptions in 2009 was USD 6.5 billion [12].

Only limited success has been achieved in lowering the prescription rate of antibiotics. One explanation could be that physicians’ prescribing rates differ in ways that are not justified by patient factors [13,14,15,16,17]. AMR can be controlled by using antibiotics appropriately. Expenses associated with morbidity and mortality are one aspect of the economic burden with inappropriate treatment. Most ARTI cases are treated with antibiotic therapy; as such, the chosen antibiotic must be effective, safe, and cost-effective. The purpose of this study was to evaluate the prevalence and patterns of antibiotic use for ARTIs among the Saudi population, including the frequency and antibiotic prescription errors, as well as the financial impact of improper antibiotic use.

## 2. Methods

### 2.1. Study Design

This was a retrospective cross-sectional study conducted in the ED of King Abdulaziz Medical City (KAMC), Riyadh, Saudi Arabia, by reviewing patient charts for those in the ED complaining of ARTIs over six months, from January to June 2021.

### 2.2. Study Setting

This study was conducted in the ED of KAMC, a 1505-bed university-affiliated tertiary care center, accredited by the Joint Commission International.

### 2.3. Study Population

All patients admitted to the ED for ARTI during the first half of the year of 2021 were enrolled in the study. As stated, RTIs included URTIs and LRTIs. Patients were classified as pediatric, adult, or elderly: those under 15 years as “pediatric,” those 15–64 years as ‘‘adults,’’ while those 65 or more as ‘‘elders’’ or ‘‘older adults.’’ Pediatric cases were classified into less than 2 years, 2 through 6 years, and 7 through 15 years. These operational definitions were adopted as they were used in similar studies [18,19].

### 2.4. Data Collection

The following data were reviewed during ED visits in the study. Patient characteristics were demographic data, number of visits to the ED in 6 months, and health status. Antibiotic traits included name and category (i.e., penicillin, cephalosporin, macrolide, and fluoroquinolones), dose, frequency, duration of antibiotic therapy, and cost. Microbiology characteristics included type of culture collected, e.g., sputum/throat/nasal, results of culture (positive or negative), and the identified microorganism.

### 2.5. Outcome Characteristics

There were three study outcomes: effectiveness of antibiotics for treatment of ARTIs measured by recurrent visits to the ED, infections during the study period, inappropriateness of antibiotic treatment, and cost analysis. Inappropriateness of antibiotic treatment included errors in selection, dosage, frequency, and duration. Therefore, it is defined as selection of an antibiotic that is neither the drug of choice nor the alternative drug indicated, or an inappropriate dose, frequency between doses, or duration of treatment. Inappropriate dose was more or less than the recommended daily amount of the antibiotic. Inappropriate frequency was more or less than recommended. Inappropriate duration was shorter or longer than recommended. For inappropriate dose and duration, variability of ±5% was acceptable between the prescribed and recommended dose and duration; variation beyond this margin was identified as inappropriate. Each antibiotic prescription was evaluated per guidelines of the AHFS Drug Information from the American Society of Health System Pharmacists and the *Drug Information Handbook: A Comprehensive Resource for All Clinicians and Healthcare Professionals* [20,21].

### 2.6. Estimates of Treatment Cost

Only costs charged in the ED were considered for patients admitted and discharged from that unit. The total direct cost to the hospital for treatment of URTIs was analyzed. Indirect costs, such as those associated with sickness, were not included. Treatment costs did not involve medical equipment used. US dollars were used to calculate study costs. Direct treatment costs included physicians’ fees, diagnostic tests (for RTIs), and prescription drugs (antibiotics only included and over-the-counter drugs were not considered). Prescription drugs were generally free for patients.

### 2.7. Data Management and Analysis

SPSS Statistical Software (v. 22; SPSS Inc., Chicago, IL, USA) was used for data entry and analysis. Bivariate analysis using Pearson’s chi-square test (χ^2^) was carried out for categorical data such as age, sex, and drug. Inappropriate antibiotic prescriptions were determined as the number of physician orders with one or more types of errors divided by the total number of prescriptions multiplied by 100. The prevalence of errors (selection, dose, frequency, and duration) was deemed discordant by dividing the number of errors into the number of antibiotic prescriptions, then multiplying by 100. For cost, data were summarized as mean ± SD or median (range) for continuous variables, with numbers and percentages for categorical variables when appropriate: χ^2^ was used for these variables. For all statistical tests, a value of *p* < 0.05 was statistically significant.

### 2.8. Ethical Issues

This study was approved by the Research Committee of King Abdullah International Medical Research Center (KAIMRC), King Saud Bin-Abdulaziz University for Health Sciences (NRC22R/460/09). Patient informed consent to review their medical files was not required and waived by the Research Committee: this was a retrospective study without communication with patients. Patients’ privacy and data confidentiality were secured by the principal investigator.

## 3. Results

### General Characteristics and Prevalence of RTIs

**Figure 1:**
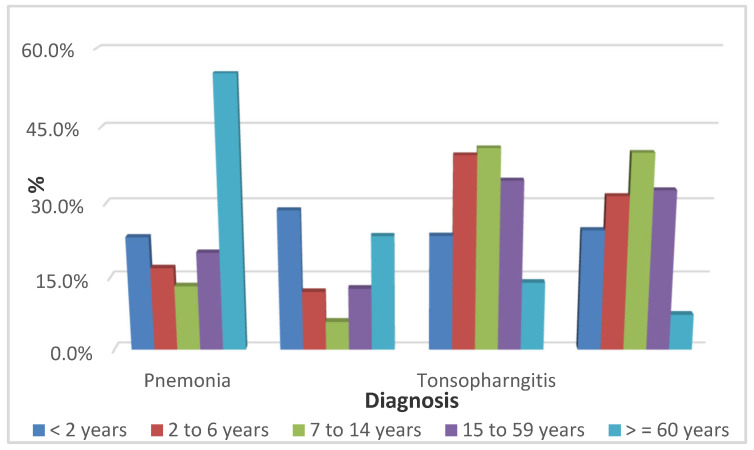
Distribution of antibiotics prescribed in different age groups by diagnosis.

**Figure 2:**
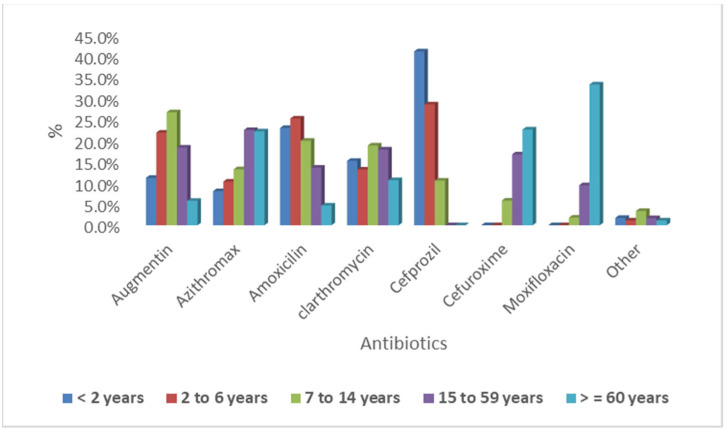
Distribution of antibiotics prescribed by age category.

The overall prevalence of ARTI was 21.8% of total visits to the ED (Table 1). There were 3185 patients admitted to the ED for ARTI, both pediatric (1428, 44.8%) and adults (1757, 55.2%). Males and females represented 51.1% and 48.9%, respectively. During the study period, 981 out of 1428 (68.7%) of pediatric cases complained of URTI, while 447 out of 1428 complained of LRTI. The highest age group of pediatric cases with URTIs was 2 to 6 years, followed by 7 to 14 years, and lastly less than 2 years. The highest age group of pediatric cases with LTRIs was 2 to 6 years, followed by less than 2 years, and lastly 7 to 14 years. The cultures were ordered for 401 (12.6%) of patients, among which 132, 15, 31, and 223 presented with throat, sputum, nasopharyngeal, and other infections. The result of culture was as follows: 56 out of 401 (14%) were positive and 345 out 401 (86%) were negative. Group A streptococcus represented 32 of 56, respiratory syncytial virus (RSV) represented 15 of 56, while Streptococcus pneumoniae represented 2 of 56, and Group C streptococcus represented 4 of 56. Most patients (79.8%) had one course of antibiotics during the study, followed by 16% with two courses, and 3.9% with three courses. In Table 1 and Figure 1 and Figure 2, penicillin (pediatrics 43%, adults 26%), cephalosporin (pediatrics 29%, adults 19%), and macrolide (pediatrics 26%, adults 38%) were the three main ATBs prescribed for both age groups. There were significant differences in each of the three categories, with *p* values at 0.001, 0.001, and 0.010, respectively. Most patients were given broad-spectrum antibiotics (73.2%).

## 4. Clinical Outcomes

During the study period, 81.5% had one episode of infection and completely recovered (Table 2). As presented in Table 2, 53% of antibiotics (pediatrics 35% and adults 67%) were prescribed inappropriately, where error in dosing represented 22.1%, error in frequency represented 3.7%, error in duration represented 32.2%, and error in selection represented 3.3%. In pediatrics, dosage and duration errors were predominant (*p* < 0.001 and *p* < 0.0001, respectively), while in the adults, error in selection was significantly higher (*p* = 0.001).

Binary logistic regression and age stratification were used. Cephalosporin prescriptions vs. penicillin prescriptions and broad-spectrum ATBs in adults were significant predictors of inappropriate prescriptions. For one episode of RTI, the mean cost was USD 134.56 (95% CI USD 132.94–USD 136.17). Treatment of RTI was more costly in adults (59.2%), LRTI and those using broad-spectrum antibiotics (86.5%) (Table 3). There were statistically significant associations for sex, age, spectrum of antibiotics, category of antibiotic, and inappropriate cost (Table 4).

## 5. Discussion

This study provides information in three areas: the prevalence of ARTI among ED visits, effectiveness of treatment and prevalence of inappropriate prescribing of antibiotics, and cost of treatment, including cost of inappropriate treatment of ARTI. Inappropriate testing and treatments result in significant overspending in managing uncomplicated illnesses. ARTI is one of the most common diagnoses seen in EDs in the US [22]. Between 1995 and 2000, there was an average of 8.5 million annual ARTI visits to EDs [22], representing about 8% of all ED visits [22]. In our study, ED visits due to ARTI were higher, thus representing 21.8% during the study period. In line with previous research [23,24,25,26,27] male gender was predominant in our study.

In the current study, the prevalence of ARTI in pediatrics affected those age 7 was 30.8%, slightly higher than the prevalence in a previous study, in which RTI prevalence in children less than 5 was 24% [24]. A study of Al Mukarramah showed that the rate of ARTI in children <5 years was 39.4% [23], which was higher than our findings. Several reports showed an incidence of RTI among children <5 years ranging from 30% to 40% [25,28]; yet, others found an incidence of 83.2% [29].

As per previous studies [23,25], the prevalence of URTIs in our study was higher in pediatric cases than LRTIs. In contrast to the study by Safraz [30], our study and others showed pediatrics had LRTIs, such as pneumonia, bronchitis, and bronchiolitis [23,24,25]. The results of this study showed that antibiotics prescribed for 95.2% of visits for ARTI were higher than those previously reported [22,23]. The current study found that penicillin (34.2%), followed by macrolide (32.9%) and cephalosporin (23.8%) were the most common ATBs prescribed for patients with ARTIs. These results match those observed in earlier studies [23,29].

The results of the current study revealed that 53% of antibiotic prescriptions (pediatrics 35%; adults 67%) were given inappropriately. We observed that adult patients were considerably more likely to have been prescribed improper antibiotics than pediatric patients. This corroborates other studies that found the same pattern with all inappropriately prescribed antibiotics in EDs, not limited to ARTI [29]. The causes of this are not apparent and need more investigation. It could be due to lack of comorbid conditions in children, or that judgments about antibiotics for them are simpler. Yet, this population may be reluctant to receive prescribed drugs.

In this study, antibiotics were inappropriately prescribed due to error in duration, 32.2%, followed by error in dosing, 22.1%, error in frequency 3.7%, and error in selection 3.3%. Overprescribing increased risk of unnecessary antibiotic-related adverse events, opportunistic infections, and AMRs [31,32]. This necessitates antimicrobial stewardship (ASM) specifically designed for the ED setting to prevent unnecessary antibiotic exposure. As suggested by a previous study [29], potential AMS approaches involve an increased number of ED-based clinical pharmacists, implementing computerized prescribing platforms with clinical decision support tools, with ED-specific antibiograms [33,34]. Antibiotic resistance, needless side events, and unsuccessful treatment outcomes can result from improper antibiotic dosing.

It should be noted that the ED site is challenging and the pattern of ATB prescribing in the ED may vary more than other outpatient settings as described by previous reports [35]. In that study, there was a less significant overall decrease in ATB prescribing from baseline to intervention periods, and there were fewer classes of antibiotics that had substantial decline in use when compared with primary care outpatient settings. Given these considerations, further future study including outpatient settings is required.

This study was limited to the ED, and our conclusions might not apply to other populations and settings in Saudi Arabia. Thus, the generalizability may also be limited by the study’s single-center design and the results of this study may not be broadly applicable to other centers or patient populations. We did not differentiate between antibiotics prescribed at discharge and those given in the ED, a major limitation of this study. Moreover, it is possible that there were other variables linked to antibiotics prescribed for ARTIs in ED visits, but they were not investigated in this study. All these factors had an impact on whether antibiotics were prescribed.

## Figures and Tables

**Table 1:** Bivariate analysis of patient characteristics and antibiotic prescriptions compared by age groups.

Characteristics	Sample N (%) *n* = 3185	Less Than 2 Years *n* (%) 349 (11)	2 to 6 Years *n* (%) 632 (19.8)	7 to 14 Years *n* (%) 447 (14)	15 to 59 Years *n* (%) 1305 (41)	≥60 Years *n* (%) 452 (14.2)	χ^2^, *p*-Value
Sex							χ^2^ = 60.433, *p* < 0.000
Male	1629 (51.1)	199 (57)	367 (58.1)	271 (60.6)	577 (44.2)	215 (47.6)	
Female	1556 (48.9)	150 (43)	265 (41.9)	176 (39.4)	728 (55.8)	237 (52.4)	
Health Status							χ^2^ = 567.8, *p* < 0.000
Healthy	2526 (79.3)	349 (100)	631 (99.8)	406 (90.8)	902 (69.1)	238 (52.7)	
Non healthy	659 (20.7)	0	1 (0.2)	41 (9.2)	403 (30.9)	214 (47.3)	
Diagnosis							χ^2^ = 443.45, *p* < 0.000
URTI	1953 (61.3)	171 (49)	449 (71)	361 (80.8)	876 (67.1)	96 (21.2)	
LRTI	1232 (38.7)	178 (51)	183 (29)	86 (19.2)	429 (32.9)	356 (78.8)	
Request Culture at ED							χ^2^=123.8, *p* < 0.000
Yes	401 (12.6)	87 (24.9)	120 (19)	63 (14.1)	79 (6.1)	52 (11.5)	
No	2784 (87.4)	262 (75.1)	512 (81)	384 (85.9)	1226 (93.9)	400 (88.5)	
Result of Culture							χ^2^ = 136.89, *p* < 0.000
Positive	56 (14)	19 (21.8)	15 (12.5)	11 (17.4)	8 (10.1)	3 (5.8)	
Negative	345 (86)	68 (78.1)	105 (87.5)	52 (82.5)	71 (89.9)	49 (94.2)	
No. of Antibiotics within 3 Months							
1	2534 (79.6)	285 (81.7)	519 (82.1)	406 (90.8)	1029 (78.9)	295 (65.3)	χ^2^ = 152.7, *p* < 0.000
2	525 (16)	55 (15.8)	107 (16.9)	37 (8.3)	208 (15.9)	118 (26.1)	
3	126 (3.9)	9 (2.6)	6 (0.9)	4 (0.9)	68 (5.3)	39 (8.6)	
Antibiotic Group							χ^2^ = 666.9, *p* < 0.000
Penicillin	1090 (34.2)	120 (34.4)	297 (47)	210 (74)	415 (31.8)	48 (10.6)	
Cephalosporin	757 (23.8)	148 (42.4)	186 (29.4)	85 (19)	233 (17.9)	105 (23.2)	
Macrolide	1049 (32.9)	81 (23.2)	149 (23.6)	144 (32.2)	526 (40.3)	149 (33)	
Quinolone	288 (9)	-	-	8 (1.8)	131 (10)	149 (33)	
Antibiotic Spectrum							χ^2^ = 37.36, *p* < 0.000
Broad	2330 (73.2)	263 (75.4)	466 (73.7)	334 (74.7)	989 (75.8)	278 (61.5)	
Narrow	855 (26.8)	86 (24.6)	166 (26.3)	113 (25.3)	316 (24.2)	174 (38.5)	
Type of Treatment							χ^2^ = 212.004, *p* < 0.000
Single antibiotics	3033 (95.2)	345 (98.9)	628 (99.4)	441 (98.7)	1247 (95.6)	372 (82.3)	
Combination of antibiotics	152 (4.8)	4 (1.1)	4 (0.6)	6 (1.3)	58 (4.4)	80 (17.7)	

Abbreviations: χ^2^, Pearson chi-square test; URTI, upper respiratory tract infection; LRTI, lower respiratory tract infection; ED, emergency department.

**Table 2:** Outcome of treatment for respiratory tract infections.

Characteristics	Sample *n* (%) *n* = 3185	Less Than 2 Years *n* (%) 349 (11)	2 to 6 Years *n* (%) 632 (19.8)	7 to 14 Years *n* (%) 447 (14)	15 to 59 Years *n* (%) 1305 (41)	≥60 Years *n* (%) 452 (14.2)	χ^2^, *p*-Value
Effectiveness of Antibiotics							χ^2^ = 27.883, *p* < 0.000
Complete recovery	2597 (81.5)	283 (81.1)	512 (81)	401 (89.7)	1055 (80.8)	346 (76.5)	
Recurrent infection	588 (18.5)	66 (18.9)	120 (19)	46 (10.3)	250 (19.2)	106 (23.5)	
Frequency of Episodes in 3 Months							χ^2^ = 83.955, *p* = 0.000
Once	2597 (81.5)	283 (81.1)	512 (81)	401 (89.7)	1055 (80.8)	346 (76.5)	
Twice	466 (14.6)	51 (14.6)	110 (17.4)	40 (8.9)	187 (14.3)	78 (17.3)	
Three	83 (2.3)	15 (4.3)	10 (1.6)	6 (1.3)	28 (2.1)	24 (5.3)	
Three or more	39 (1.2)	0	0	0	35 (2.7)	4 (0.9)	
Appropriate Prescribing of Antibiotics							
Inappropriate	1689 (53)	109 (31.2)	187 (29.6)	203 (45.4)	887 (68)	303 (67)	χ^2^ = 368,936, *p* < 0.000
Type of Error							
Dose error	705 (22.1)	171 (49)	313 (49.5)	116 (26)	70 (5.4)	35 (7.7)	χ^2^ = 692.269, *p* < 0.000
High dose	317 (10)	63 (18.1)	119 (18.8)	44 (9.8)	58 (4.4)	33 (7.3)	
Low dose	145 (4.6)	44 (12.6)	58 (9.2)	30 (6.7)	12 (0.9)	2 (0.4)	
Other	242 (7.6)	64 (18.3)	136 (21.5)	42 (9.4)	0	0	
Frequency error	117 (3.7)	22 (6.3)	13 (2.1)	11 (2.5)	50 (3.8)	21 (4.6)	χ^2^ = 23.836 *p* < 0.002
High frequency	86 (2.7)	14 (4)	10 (1.6)	8 (1.8)	34 (2.6)	20 (4.4)	
Low frequency	31 (1)	8 (2.3)	3 (0.5)	3 (0.7)	16 (1.2)	1 (0.2)	
Duration error	1027 (32.2)	135 (38.7)	268 (42.4)	186 (41.2)	341 (26.1)	99 (21.9)	χ^2^ = 97.216 *p* = 0.000
Long	61 (1.9)	2 (0.6)	35 (5.5)	8 (1.8)	12 (0.9)	4 (0.9)	
Short	700 (22)	118 (33.8)	171 (27.1)	125 (28)	243 (18.6)	43 (9.5)	
Other	266 (8.4)	15 (4.3)	62 (9.8)	51 (11.4)	86 (6.6)	52 (11.5)	
Selection error	9 (3.3)	19 (2.2)	7 (2.2)	35 (2.4)			
Cost Analysis							
Mean ± SD	121.34 ± 34.972	122.45 ± 37.601	115.13 ± 31.704	114.12 ± 30.559	117.92 ± 32.508	146.17 ± 37.418	
*p* value	<0.0001						
95% CI		118.49–126.41	112.65–117.61	111.28–116.96	116.16–119.69	116.16–119.69	121.34–34.972
Cost of inappropriate antibiotic (USD) Mean ± SD	21.89 ± 12.63	25.12 ± 13.76	25.88 ± 12.01	24.73 ± 13.25			
95% CI	19.77–24.02	23.96–27.81	23.77–26.46	23.73–25.72			
% Cost of inappropriate antibiotics	47.4	47.1	48.2	47.5			

Abbreviations: χ^2^, Pearson chi-square test; 95% CI, confidence interval.

**Table 3:** Bivariate analysis of inappropriate antibiotic prescriptions as compared by RTI.

Characteristics	Sample *n* (%) *n* = 3185	Pneumonia *n* (%) 759	Other LRTI *n* (%) 154	COPD *n* (%)34	Bronchitis *n* (%) 146	Bronchiolitis *n* (%) 140	Tonsillopharyngitis *n* (%) 1029	Other URTI *n* (%) 880	Sinusitis *n* (%) 43	χ^2^, *p*-Value
Sex										χ^2^ = 18.85, *p* < 0.000
Male	1629 (51.1)	354 (46.6)	83 (53.9)	13 (38.2)	69 (47.3)	83 (59.3)	561 (54.5)	442 (50.2)	24 (55.8)	
Female	1556 (48.9)	405 (53.4)	71 (46.1)	21 (61.8)	77 (52.7)	57 (40.7)	468 (45.5)	438 (49.8)	19 (44.2)	
Health Status										χ^2^ = 567.8, *p* < 0.000
Healthy	2526 (79.3)	349 (100)	631 (99.8)	406 (90.8)	902 (69.1)	238 (52.7)				
Unhealthy	659 (20.7)	0	1 (0.2)	41 (9.2)	403 (30.9)	214 (47.3)				
Type of Treatment										χ^2^ = 392.5, *p* < 0.000
Single antibiotic	3044 (95.6)	629 (82.9)	150 (97.4)	30 (88.2)	145 (99.3)	140 (100)	1027 (99.8)	880 (100)	43 (100)	
Combined antibiotics	141 (4.4)	130 (17.1)	4 (2.6)	4 (11.8)	1 (0.7)	-	2 (0.2)	-	-	
Request for sputum culture in ED										χ^2^ = 123.8, *p* < 0.000
Yes	401 (12.6)	87 (24.9)	120 (19)	63 (14.1)	79 (6.1)	52 (11.5)				
No	2784 (87.4)	262 (75.1)	512 (81)	384 (85.9)	1226 (93.9)	400 (88.5)				
Results of Culture										χ^2^ = 136.89, *p* < 0.000
Positive	56 ()	19 (21.8)	15 (12.5)	11 (17.4)	8 (10.1)	3 (5.8)				
Negative	345 ()	68 (78.1)	105 (87.5)	52 (82.5)	71 (89.9)	49 (94.2)				
Antibiotic Group										χ^2^ = 1885.3, *p* < 0.000
Penicillin	1090 (34.2)	48 (6.3)	88 (57.1)	5 (14.7)	24 (16.4)	28 (20)	308 (29.9)	573 (65.1)	16 (37.2)	
Cephalosporin	757 (23.8)	267 (35.2)	20 (13)	13 (38.2)	34 (23.3)	74 (52.9)	112 (10.9)	224 (25.5)	13 (30.2)	
Macrolide	1049 (32.9)	169 (22.3)	45 (29.2)	15 (44.1)	82 (56.2)	38 (27.1)	609 (59.2)	81 (9.2)	10 (23.3)	
Quinolone	288 (9)	274 (36.1)	1 (0.6)	1 (2.9)	6 (4.1)	-	-	2 (0.2)	4 (9.3)	
Antibiotic Spectrum										χ^2^ = 37.36, *p* < 0.000
Broad	2330 (73.2)	263 (75.4)	466 (73.7)	334 (74.7)	989 (75.8)	278 (61.5)				
Narrow	855 (26.8)	86 (24.6)	166 (26.3)	113 (25.3)	316 (24.2)	174 (38.5)				

**Table 4:** Total cost of treatment for respiratory tract infections.

Characteristics	*n*	% *n*	Mean	SD	Median	Minimum	Maximum	Sum	% Total	SE	*p*-Value	Lower 95% CI	Upper 95% CI
Sex													
Male	1629	51.1%	120.57	34.381	102.99	80	273	196416	50.8%	0.852	0.207	118.90	122.25
Female	1556	48.9%	122.14	35.574	104.56	80	274	190050	49.2%	0.902		120.37	123.91
Age											<0.0001		
<2 years	349	11.0%	122.45	37.601	104.56	83	224	42736	11.1%	2.013	<0.0001	118.49	126.41
2 to 6 years	632	19.8%	115.13	31.704	95.07	83	206	72763	18.8%	1.261		112.65	117.61
7 to 14 years	447	14.0%	114.12	30.559	101.71	80	235	51013	13.2%	1.445		111.28	116.96
15 to 59 years	1305	41.0%	117.92	32.508	102.99	80	274	153887	39.8%	0.900		116.16	119.69
>=60 years	452	14.2%	146.17	37.418	151.81	80	243	66068	17.1%	1.760		142.71	149.63
Age Category											<0.0001		
Adult	1757	55.2%	125.19	36.012	110.61	80	274	219955	56.9%	0.859		123.50	126.87
Pediatric	1428	44.8%	116.61	33.051	101.71	80	235	166512	43.1%	0.875		114.89	118.32
Category Diagnosis											<0.0001		
URTI	1953	61.3%	100.84	17.810	93.25	80	207	196948	51.0%	0.403		100.05	101.63
LRTI	1232	38.7%	153.83	30.619	153.27	83	274	189518	49.0%	0.872		152.12	155.54
Category of Tx											<0.0001		
Pneumonia	759	23.8%	164.09	25.978	163.95	85	274	124543	32.2%	0.943		162.24	165.94
LRTI	154	4.8%	147.73	19.618	147.16	85	235	22751	5.9%	1.581		144.61	150.85
COPD	34	1.1%	138.58	28.281	146.58	87	195	4712	1.2%	4.850		128.71	148.45
Bronchitis	146	4.6%	119.00	27.681	112.18	84	227	17374	4.5%	2.291		114.47	123.53
Bronchiolitis	140	4.4%	143.62	35.323	145.61	83	224	20107	5.2%	2.985		143.62	137.72
URTI	880	27.6%	97.73	14.322	92.28	80	159	86005	22.3%	0.483		96.79	98.68
Tonsopharyngitis	1029	32.3%	103.43	20.074	93.25	80	207	106432	27.5%	0.626		102.20	104.66
Sinusitis	43	1.4%	105.66	17.330	98.48	83	164	4543	1.2%	2.643		100.32	110.99
Result of Culture											<0.0001		
Not requested	2784	87.4%	114.68	29.992	101.87	80	273	319273	82.6%	0.568		113.57	115.80
Negative	345	10.8%	167.96	33.046	177.63	92	274	57947	15.0%	1.779		164.46	171.46
Positive	56	1.8%	165.11	25.012	155.04	136	224	9246	2.4%	3.342		158.41	171.81
Antibiotic Group											<0.0001		
Cephalosporin	757	23.8%	135.91	35.755	136.37	80	243	102883	26.6%	1.300		133.36	138.46
Macrolide	1049	32.9%	113.66	27.672	102.99	80	227	119230	30.9%	0.854		111.98	115.34
Penicillin	1090	34.2%	105.19	24.762	94.85	80	200	114661	29.7%	0.750		103.72	106.67
Quinolone	288	9.0%	172.01	27.065	175.21	83	274	49539	12.8%	1.595		168.87	175.15
Antibiotic Spectrum											<0.0001		
Broad	2330	73.2%	120.98	31.852	106.40	80	243	281884	72.9%	0.660		119.69	122.27
Narrow	855	26.8%	122.32	42.330	94.85	80	274	104583	27.1%	1.448		119.48	125.16
Type of Course											<0.0001		
Monotherapy	3033	95.2%	119.69	34.418	102.99	80	274	363025	93.9%	0.625			
Combination	152	4.8%	154.22	29.323	149.20	85	220	23442	6.1%	2.378			
Recurrent Disease											<0.0001		
Once	2597	81.5%	120.09	34.431	102.99	80	274	311884	80.7%	0.676		118.77	121.42
Twice	466	14.6%	125.26	36.392	111.40	80	228	58370	15.1%	1.686		121.94	128.57
Three	83	2.6%	130.80	37.227	141.87	83	211	10856	2.8%	4.086		122.67	122.67
More than three	39	1.2%	137.35	39.240	146.58	83	238	5357	1.4%	6.283		124.63	124.63
Total	3185	100.0%	121.34	34.972	102.99	80	274	386467	100.0%	0.620		120.12	122.55

Std. Error of Mean, Std. Deviation, Confidence Interval.

## Data Availability

Not applicable.
